# The Impact of Ankle-foot Orthoses on Balance in Older Adults: a Scoping Review

**DOI:** 10.33137/cpoj.v4i1.35132

**Published:** 2021-01-04

**Authors:** J.L Laidler

**Affiliations:** Department of Rehabilitation Therapy, Faculty of Health Sciences, Queen’s University, Kingston, Canada.

**Keywords:** Ankle-Foot Orthosis, AFO, Older Adult, Aged, Balance, Postural Balance, Orthosis, Scoping Review, Fall

## Abstract

**BACKGROUND:**

Balance impairment is a contributing factor to falls. Falls are a leading cause of injury and death in older adults. An ankle-foot orthosis (AFO) is a device that can be prescribed as an intervention to help individuals with compromised balance to ambulate safely.

**OBJECTIVE:**

The purpose of this review was to investigate the role ankle-foot orthoses have in affecting balance in community-dwelling older adults.

**METHODOLOGY:**

A scoping review was conducted searching MEDLINE, CINAHL, EMBASE, and REHABDATA databases to obtain the appropriate literature to meet the following criteria: 1) quantitative research design; 2) studies with participants over age 65; 3) studies with participants with drop-foot or sensory deficits in the lower extremity; 4) the treatment intervention was unilateral or bilateral AFOs; 5) the outcome measure was balance or stability. The retrieved articles were assessed based on the internal validity, external validity, objectivity, and reliability of the study design and the interpretation of results.

**FINDINGS:**

11 articles were identified that met the inclusion criteria. Four major themes emerged in the analysis about the impact that ankle-foot orthoses have on balance in older adults: (1) AFOs improved lateral stability, (2) AFOs improved balance under static conditions, (3) AFOs provided a reduction in postural sway and (4) AFOs increased walking speed in community-dwelling older adults.

**CONCLUSIONS:**

The evidence from the findings of the review indicate that ankle-foot orthoses have a generally positive affect on balance in older adults. Clinicians can consider the ankle-foot orthosis an effective intervention that can improve balance in some older adult patient populations.

## INTRODUCTION

Falls are the leading cause of injury among older adults in Canada and are the third leading cause of death after cancer and heart disease.^1^ One in three adults over age 65,^2^ and one in two adults over age 80,^3^ experience at least one fall annually. Falls are associated with high morbidity and mortality, and poor health outcomes.^4,5^ Older adults experience more falls and have a high susceptibility to injury, making falls a significant public health issue. Age-related physiological changes coupled with a higher prevalence of comorbidities^6,7^ can result in older adults experiencing fractures, hospitalization, or early admission to a long-term care facility.^8^ Greater than 70% of falls in the community occur in the home, due to both predisposing and situational risk factors.^5^ With increasing numbers of older adults wishing to stay in their homes and ‘age-in-place’, falls are of increasing concern.

Issues in balance control have been identified as a strong risk factor for falls.^4^ Age-related sensory and musculoskeletal changes play a large role in affecting balance in older adults.^5,8,9^ Decreased muscle mass is notable in aging and can lead to weakness resulting in inactivity, decreased balance control, gait deviations and instability during ambulation, and a lessened quality of life.^7,10–12^ Age-related physiological changes can co-occur with the development of chronic condition(s), further compromising balance depending on underlying issues, and the strategies used to help manage them. Ankle-foot orthoses (AFOs) are often prescribed for managing pathologies that affect typical functioning of the ankle joint in stability, positioning, and pressure distribution.^13^ An ankle-foot orthosis is a brace worn on the lower leg to hold the foot and ankle in position, defined by the International Society for Prosthetics and Orthotics as an “externally applied device used to modify the structural and functional characteristics of the neuromuscular and skeletal system.”^14^ AFOs have been accepted as a treatment to address balance impairment, proper gait parameters, and safe ambulation for people with conditions such as stroke, peripheral neuropathy, multiple sclerosis, cerebral palsy and others.^13^ Evidence exists to support the use of AFOs to improve ambulation and joint alignment, the next step is to determine what evidence exists regarding the effect of AFOs on balance.

The objective of this review is to investigate the influence of AFOs during static and dynamic balance in older adults. The research question will be: What role do ankle-foot orthoses play in affecting balance in community-dwelling older adults?

## METHODOLOGY

A scoping review was conducted to identify the existing relevant literature available on the subject and to evaluate the research findings. Scoping reviews examine the range and nature of research literature in a specific subject area, and commonly aim to identify gaps in the existing literature to determine the value of undertaking a full systematic review.^15^ The research question used to guide the review was “What role do ankle-foot orthoses play in affecting balance in community-dwelling older adults?” The definition of an older adult was men or women aged 65 or older. Community-dwelling older adults were considered as those living in their own homes, not in institutions such as hospitals or long-term care.

### Search strategy

An electronic database search was conducted using four databases: MEDLINE, CINAHL, EMBASE, and REHABDATA. These databases were selected because they contain literature pertaining to the health sciences, and they index the main journals that contain information related to healthcare and rehabilitation. To supplement these searches, a hand search of reference lists of retrieved articles was also conducted to identify potentially relevant studies.

**Figure 1:**
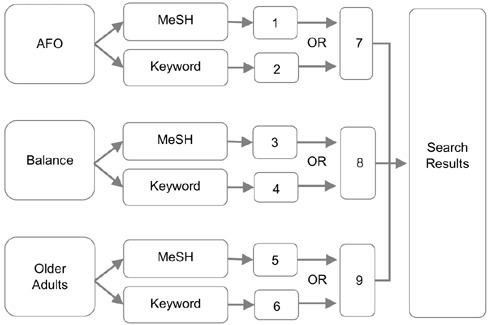
Example database search strategy. Each number represents the database search performed and the results retrieved for each MeSH term and keyword (1-6), the searches combining the corresponding MeSH term and keyword (7,8,9), and the final search results combining all of the terms involved.

A combination of keywords and MeSH terms were used to conduct the search. The search terms were as follows: orthotic device/orthotic brace/ankle-foot orthotic/AFO; balance/postural balance; aged/older adults. MeSH terms and corresponding keywords were combined in searches with ‘AND’ or ‘OR’ to ensure the articles retrieved contained all relevant terms. Figure 1 illustrates the search strategy undertaken in each database (Appendix (A)) for detailed database searches). Literature from January 1990 to February 2020 were included in the search based on discussions with professionals in the field, as well as through database searches, which determined that limited literature existed on this topic prior to 1990.

### Article screening and selection process

Titles and abstracts of retrieved articles were screened for relevance to the research question based on the following criteria: **1)** studies had a quantitative design; **2)** participants were older adults over the age of 65; **3)** participants had some degree of drop-foot or a sensory deficit in their lower extremity; **4)** the treatment intervention was unilateral or bilateral ankle-foot orthoses; **5)** the outcome measure was that of balance or stability. The full text of the remaining studies that were identified as relevant were screened for eligibility based on inclusion and exclusion criteria.

Studies were included if they met the following inclusion criteria: **1)** participants were community-dwelling older adults, aged 65 and over; **2)** study incorporated the use of unilateral or bilateral AFOs, regardless of design or fabrication material; **3)** study described the effect of the AFO(s) on balance; **4)** study was written in English. Studies were excluded from consideration if **1)** the AFO(s) had a mechanical/electrical component; **2)** patients had partial foot amputations; **3)** participants were in hospital or long-term care. These criteria were selected to ensure that the most appropriate patient population and study conditions were included in the analysis to address the research question. Where possible, in studies with mixed-aged samples of participants under the age of 65, only data from those 65 and over were considered. Each article was screened for eligibility based on whether it met these criteria, and whether the title and abstract contained the relevant content to address the research question to warrant full-text review. The reference management software EndNote version X9.2 (EndNote, Clarivate Analytics, PA, USA), was used to manage the citations of the retrieved articles.

### Data extraction and analysis

Articles were selected based on their focus on the affect that AFOs have on balance in the older adult population. The full text of each article was examined by the reviewer and was assessed based on the internal validity, external validity, objectivity, and reliability of the study design and the authors’ interpretation of the results. Key information was extracted and organized into a table to display the main ideas and characteristics of each study including study aim and findings, sample characteristics, study design, AFO characteristics, balance metrics utilized, and strengths and limitations.

The quality of each of the selected articles was also critically evaluated using a series of quality appraisal questions based on a combination of appraisal tools developed by CASP^16^ and McMaster University.^17^ Quality was determined by using these questions to evaluate the methodological vigour of the study design and the soundness of the interpretation of findings presented in each article. Based on the determined quality, a grade was assigned (from 1-low to 3-high) to each study, and a quality matrix was developed based on the quality rating to determine the weight of the evidence presented in each study (Appendix (B)). Higher quality evidence was weighted greater when considering its value in addressing the research question.

Articles were analyzed based on the similarities of subject characteristics and diagnoses, balance metrics recorded, and study design. The findings of each article were examined and compared for relevancy to answering the research question, and for emergent patterns on the effects AFOs produce on balance in older adults.

## RESULTS

**Figure 2:**
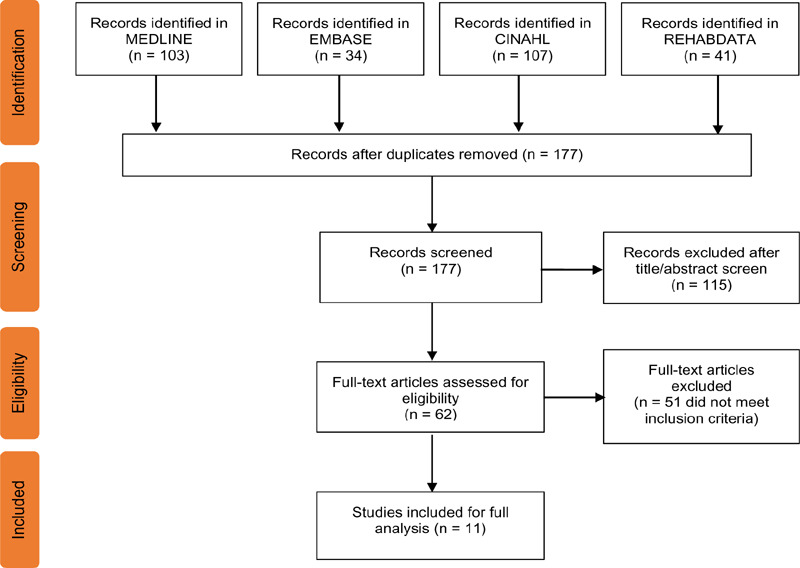
Flowchart diagram of the literature search, screen and selection process

A total of 285 studies were retrieved from the database searches, 108 of which were duplicates and were disregarded. The titles and abstracts of 177 articles were screened for relevancy to answering the research question, producing 62 articles for full-text review. After the inclusion and exclusion criteria were applied, 11 articles remained for inclusion in the scoping review (Figure 2). Hand searches of reference lists yielded only duplicate or irrelevant studies, thus did not add to the search results.

The emergent trends in the findings of each article were identified as the core themes existing in the current research literature and were the themes that were examined to help address the research question. The 11 articles included were selected because they focused on the affect that AFOs have on balance in the older adult population. Through the quality appraisal and data analysis of each article, four major themes emerged: AFOs improved lateral stability, static balance and walking speed, and provided increased postural control. The quality matrix (Appendix (B)) summarizes the quality rating and weighting of the evidence presented in each article. The summary of the main thematic ideas identified regarding ankle-foot orthoses’ affect on balance in older adults are found in Table 1. The quality rating was inputted into the final column of the thematic summary table (Table 1) and the data extraction table (Appendix (C)).

**Table 1:** Summary of the main ideas about the affect ankle-foot orthoses have on balance in older adults.

**Author, Year**	**Sample/Study Design**	**Aim of study**	**Main Themes**				**Quality Rating**
			Lateral stability	Static balance	Postural Control	Walking speed	
Bigelow & Jackson, 2014^26^	n=12 subjects with peripheral neuropathy; Pre-/post-test Quantitative Within-subjects comparison	To observe the immediate effects of AFOs on balance and gait in individuals with peripheral neuropathy.	x	x	x		3
Cakar et al., 2010^24^	n=25 subjects with post-stroke hemiplegia with spasticity; Pre-/post-test Quantitative Within-subjects comparison	To investigate the relative effect of AFOs on balance and fall risk.		x			1
Chen et al., 2008^20^	n=21 subjects with new onset stroke hemiplegia + 10 healthy subjects; Pre-/ post-test; Quantitative Cross sectional + control group comparison	To evaluate the effects of an AFO on postural stability in stroke patients with hemiplegia.		x	x		3
Chen et al., 1999^21^	n=24 hemiplegic subjects; Pre-/ post-test Quantitative Within-subjects comparison	To evaluate the effects of an AFO on static and dynamic postural stability in hemiplegic patients.	x	x			1
Dogan et al., 2011^25^	n=51 subjects with post-stroke hemiplegia; Pre-/post-test Quantitative Within-subjects comparison	To investigate whether AFOs have an effect on stair climbing, balance and mobility while improving walking parameters.			x	x	1
Nikamp et al., 2017^18^	n=33 subjects with post-stroke hemiplegia; RCT 6-month follow-up Quantitative Parallel group comparison	To study the 6-month effects of early or late provision of AFOs in stroke patients; To look at differences between groups and functional improvement overtime.			x	x	2
Simons et al., 2009^19^	n=20 post-stroke subjects with hemiplegia; Pre-/ post-test Quantitative Within-subjects comparison with washout period	To examine effects of AFOs on functional balance, static and dynamic weight bearing asymmetry, and dynamic balance control.				x	2
Wang et al., 2019^27^	n=44 non-pathologic subjects RCT Quantitative Longitudinal (6-month follow-up)	To investigate effectiveness of AFOs on balance, fear of falling, and physical activity in older adults.	x	x	x		3
Wang et al., 2005^22^	n=103 subjects with post-stroke hemiplegia; Pre-/ post-test Quantitative between group comparison	To examine the effects of AFOs on balance in patients with short and long duration hemiparesis.	x	x		x	2
Wang et al., 2007^23^	n=58 subjects with post-stroke hemiplegia; Pre-/ post-test Quantitative Within-subjects comparison	To assess changes in balance, and improvement in gait of hemiplegic subjects as a result of wearing an AFO.	x	x	x	x	2
Yalla et al., 2014^28^	n=30 subjects with diabetes and/or peripheral neuropathy; Pre-/ post-test Quantitative Within-subjects comparison	To determine the immediate effect of AFOs on balance and functional reach distance in older adults.	x	x	x		2

### General overview of study characteristics

The included studies were conducted in the Netherlands,^18,19^ Taiwan,^20–23^ Turkey,^24,25^ and the United States.^26–28^ Nine studies used a randomized pre-test/post-test design, in which the AFO condition was compared with the no AFO condition, and the order of the testing with and without the AFO was randomized. Two studies that were included were randomized control trials (RCTs).^18,27^ Records were searched from 1990 to present, and only one study was retrieved prior to 2005.^21^

Sample sizes ranged from 12^26^ to 103^22^ subjects. The studies included a sample of participants in which forty-two percent were female, while fifty-eight percent were male. The ages ranged from twenty-six to eighty-four years, with the average age being 65. Eight of the eleven included studies had patients who were recruited from outpatient rehabilitation hospital settings.^18–25^ The remaining studies recruited patients from community medical clinics or seniors support groups.^26–28^ The studies that recruited subjects from outpatient rehabilitation used samples of hemiplegic stroke patients as their subjects, and the three studies who recruited from the community setting included subjects with peripheral neuropathy or diabetes,^26,28^ or non-pathological subjects.^27^

The style of AFOs that were used varied across studies, as well as in the duration of time subjects had getting used to using them. AFO designs utilized in the studies included thermoplastic posterior leaf-spring,^18,22–24^ thermoplastic anterior leaf-spring,^20,21,27,28^ anterior-shell carbon composite,^26^ thermoplastic hinged,^25^ or varying types.^19^ Studies included both custom and prefabricated AFOs, with the majority of studies utilizing prefabricated versions. Six studies tested the immediate balance effects of AFOs on subjects who had no prior experience using one,^20,22,23,26–28^ and five studies included subjects who had sufficient practice (>4 days) or used their own AFO at study outset.^18,19,21,24,25^ Eight of the eleven studies had their subjects wear an AFO on a single side (unilaterally),^18–25^ and three studies required their subjects to wear AFOs on both sides (bilaterally).^26–28^

### Themes

#### Lateral Stability

Improved lateral stability with AFO wear emerged as a theme throughout the literature. Six studies included findings that lateral stability was improved while wearing an AFO.^21–23,26–28^ It was found that AFO wearers were able to shift their center of mass (COM) more toward the AFO side and therefore increase the lateral stability on this affected side. Anterior/posterior balance was also measured with varying affects found on whether balance was affected due to AFO wear. Some studies observed limitations to anterior/posterior balance and suggested that the AFO restricted natural ankle movement.^21–23,26^ Chen et al.^21^ evaluated the effects of AFOs on postural stability in hemiplegic patients during weight shifting using force sensors and found significant improvements in lateral weight shifting and weight bearing on the AFO side, with no improvement observed in the anterior/posterior direction. They attributed this to the ankle’s range of motion being restricted while wearing the AFO. Wang et al.^23^ found that in their study of assessing changes in balance and gait of hemiplegic subjects wearing AFOs, it was demonstrated that though AFOs restricted ankle movement, the presence or absence of wearing an AFO did not significantly impact anterior/posterior balance measures during leaning tasks. All of the studies that found a positive relationship between AFOs and increased lateral stability, found this improvement in a static condition.

#### Static Balance

Improvements in static balance as a result of wearing an AFO was the most commonly observed theme in the reviewed studies.^20–24,26–28^ Static balance was improved while wearing AFO(s) in each of these studies and was more significant than measured improvements in tests of dynamic balance. Many studies conducted trials primarily using static measures of balance, while only some included dynamic walking test conditions. All of these studies used a computer-based devices and software to attain their balance measures.

Cakar and colleagues^24^ investigated the relative effect of AFOs on balance and fall risk by comparing balance measurement outcomes within a group of stroke patients with spasticity to determine that AFOs improved balance, though they used exclusively static testing conditions. Similarly, the studies by Chen et al.,^20^ who evaluated the effects of an AFO on postural stability in stroke patients with hemiplegia, and Wang et al.,^27^ who investigated the effectiveness of AFOs on balance in older adults, also found improvements in balance while only testing in static conditions using force plate posturography and balance sensors, respectively.

#### Postural Control

Seven studies found a reduction in postural sway as a result of AFO wear.^18,20,23,25–28^ Postural sway, or postural control, was a variable measured to some capacity in all reviewed studies. In their investigation to determine the immediate effect of AFO wear on balance and functional reach in older adults, Yalla et al.^28^ found that AFOs decreased postural sway. Bigelow & Jackson^26^ found similar results in their investigation on the immediate effects of AFOs on balance and gait using force plates and clinical tests to produce findings that postural sway was reduced in static conditions, though the improvements in postural control in dynamic balance and walking conditions were more varied.

Doğan et al.^25^ and Nikamp et al.^18^ were two studies with weaker evidence to support the AFOs ability to better control postural sway. Doğan et al.^25^ investigated whether AFOs had an effect on stair climbing, balance, and mobility using functional measurement methods for which two of three functional tests showed a decrease in postural sway, and the third showed no effect. Nikamp et al.^18^ conducted a six-month follow up to their previous RCT to evaluate the effects of early or late provision of an AFO for stroke patients and to observe the differences in functional improvements overtime between groups. They observed the trend that postural balance improved overtime and did not differ between groups. The remaining studies that did not show a reduction in postural sway, did not find a negative impact of AFOs on postural sway or balance control.^19,21,22,24^

#### Walking Speed

Five studies showed AFOs produced an effect on walking speed.^18,19,22,23,25,26^ With the exception of Bigelow & Jackson,^26^ who found that AFOs decreased walking speed, the remaining studies found that AFOs increased walking speed, with effects reported on improved gait parameters as well.^22,23^ In examining the effects of AFOs on balance in patients with short-and long-duration hemiplegia, Wang et al.^22^ found that in addition to gait speed improving, cadence was also improved while wearing an AFO in their clinical tests involving gait trials.

Simons and colleagues^19^ examined the effects that AFOs have on functional balance, and static and dynamic weight bearing asymmetry using posturographic and functional clinical tests with stroke patients wearing various AFO designs. They found that walking speed was increased in all patients regardless of the style of AFO worn. There was no consistent AFO fabrication style or design used in the trials of other studies that demonstrated an increase in walking speed, only that they were all worn unilaterally. Custom-made versus prefabricated devices also had no apparent role on the balance outcomes of older adults in the included studies.

### Quality of the evidence

The articles deemed to be of the highest quality^20,26,27^ were found to have strong objectivity and reliability, while the articles found to be of the lowest quality^21,24,25^ were found to have poor external validity. The evidence presented in the examined articles was generally found to be of moderate quality. Randomized control trials were considered to be at the top of the hierarchy of evidence,^29^ though only two were found to meet the inclusion criteria for the review.^18,27^ Most authors randomized the orthotic conditions during pretest/post-test trials so that the participants could act as their own control, while only three studies actually included distinct comparison groups.^18,20,27^ The Nikamp and colleagues^18^ study was an extension of their previous RCT involving inpatients, to investigate the six-month effects of their intervention. Though this study had elements of the strength of an RCT, it was not considered to be as high quality in its ability to answer this review’s research question. The sample sizes in the reviewed studies were small, and all studies had under 60 participants except for one.^22^

Through the quality appraisal procedure, the studies by Wang et al.,^27^ Chen et al.,^20^ and Bigelow & Jackson^26^ were considered to have the most procedurally sound designs and be the highest quality of the studies reviewed. These studies found that AFOs had an effect on balance by decreasing postural sway and improving balance under static conditions. In addition to balance improvements in these two areas, Bigelow & Jackson^26^ and Wang et al.^27^ also found significant improvements in lateral stability as a result of wearing an AFO. The trend that AFOs increase walking speed came from articles that were identified as moderate or low quality.

## DISCUSSION

The aim of this scoping review was to investigate the role ankle-foot orthoses play in affecting balance in community-dwelling older adults. Most studies found that with AFO wear postural control was improved, especially in the lateral direction. It was also demonstrated that AFOs improved static standing stability, that did not necessarily transfer into improved stability in dynamic conditions, such as walking. The trends observed from the findings of these articles provided some insight into the role that AFOs play on affecting balance in older adults.

### Main findings

### Improved Lateral Stability

Lateral stability was widely found to improve amongst AFO wearers in this review. The findings by Wang et al.^22^ confirmed earlier findings by Chen et al.,^21^ that through the use of an AFO, the body’s limit of lateral stability is increased, and subjects were able to bear more weight through their hemiplegic side. Wang et al.^23^ reconfirmed this finding in their 2007 investigation on changes in balance while wearing an AFO, which again showed the AFOs ability to improve balance through increasing lateral stability.

Age-related declines in reaction time and physical strength can lead to impaired postural control in mobility. In a study on age-related differences in lateral stability, King, Akula, & Luchies^30^ found that compared to younger subjects, older individuals generated a larger force to recover balance to account for reduced lateral stability. Age-related muscle weakness can affect an older adult’s ability to produce this response for balance recovery. Impairments in balance control leave older adults more susceptible to falls, which are a major health issue, and a cause for injury and loss of independence in this population.^31^

Many of the reviewed studies acknowledge the lack of anterior/posterior balance improvement with AFO wear and attribute it to restrictions imposed on the ankle by the AFO.^21–23,26^ Rigid AFOs decrease ankle range of motion, which Yalla and colleagues^28^ speculate could increase risk for falling. However, Wang et al.^23^ report that though the AFO did restrict ankle movement, it did not significantly impact balance. This is supported by the investigation by Chen et al.,^20^ on ankle strategies in balance responses, which found that despite the rigid ankle, patients were still able to elicit ankle strategies to maintain postural control while wearing AFOs. Further investigation on the role AFO ankle rigidity plays on balance is necessary.

### Improved Static Balance

Improved balance during static standing conditions was the most commonly observed theme in the literature in this review. Wang et al.^22^ examined stroke patients wearing AFOs under static standing conditions and found that balance improved. In reaching during static standing, Yalla et al.^28^ found that the AFOs improved static balance. In interpreting these findings, it is important to take into account that many studies in the review only measured an AFOs affect on balance during static, rather than dynamic conditions. Cakar et al.,^24^ for example, found that AFOs improved balance in spastic stroke patients under static conditions, however they did not include dynamic measures of balance in their study.

Dynamic balance was investigated in a study by Shearin, Smith, Querry, & McCain^32^ to assess individuals’ ability to attend to external demands requiring modifications to balance while walking. The results of this study found that dynamic balance was improved in individuals wearing an AFO. This finding was not validated in this review, and only one study^23^ demonstrated positive effects of AFOs on balance in a dynamic situation. As well, Simons et al.^19^ found that the AFO had no effect on either static or dynamic balance. Further investigation is required to determine the AFOs role during dynamic balance.

### Improved Postural Control

AFOs were shown to affect postural control in this review. To maintain stability during standing, the body’s center of mass (COM) must be positioned over the feet - the base of support (BOS).^33^ Even during quiet standing in healthy individuals, the COM experiences sway, though it is minimized when balance is proficient.^34^ Moving the COM outside of the BOS leads to instability, which when combined with age-related changes in strength and reaction times,^31^ can cause an increased frequency of falls.^35^

Stability and balance are significantly impacted when proprioception is impaired. Multiple studies indicated the role that AFOs play in augmenting proprioceptive feedback on the lower leg and foot when deficits exist.^20–22,26–28^ In an investigation on the AFOs ability to provide sensory cues, Aruin & Rao^36^ found that AFOs can substitute for the lack of proprioceptive feedback in sensation impaired limbs to improve the postural responses. Yalla et al.^28^ suggested that wearing an AFO promotes proprioceptive feedback through stimulating cutaneous receptors. This is especially important for patients with peripheral neuropathy, and other pathologies that impair sensation. Malas^37^ points to the important considerations this requires from orthotists when fitting an AFO to ensure that pressure is properly distributed, enabling feedback to optimize stability.

### Increased Walking Speed

In the present review, all studies that reported improved walking speed contained a sample of hemiplegic stroke patients. Since walking speed and cadence usually decrease in hemiparetic gait patterns,^38^ this finding suggests that the AFO has a regulating effect on the gait patterns in this population. Wang et al.^23^ attribute the increased walking speed to improved balance control in the affected leg that occurs as a result of wearing an AFO.

In a study on the impact AFO design has on gait parameters in stroke patients, Tyson & Thorton^39^ reported that hinged-AFOs better improved walking speed. Pardo, Galen, Gahimer, & Goldberg^40^ found similar results in their examination of hinged-AFOs effect on walking speed. In this review, only Doğan et al.^25^ used a hinged-AFO design, so this cannot explain the improvements observed. The studies that found improvements in walking speed with AFOs were of moderate quality, so further investigation into this effect is required.

The style of AFOs worn in the different studies in this review were variable, with no consistent observations made on AFO style and balance. Pardo et al.^40^ investigated the outcomes of balance tasks in individuals wearing custom and non-custom (prefabricated) AFOs. The results indicated there was no difference in balance outcomes between custom-made and prefabricated devices. These findings complement the observation in this review, that outcomes on balance were not linked to custom-fabricated devices.

### Limitations

The primary limitation of this review was that only one author was involved in the quality appraisal process of the included studies. This may have introduced some reporting bias, and could have been strengthened had additional reviewers been involved in the process. The small number of studies included in the review and that all of the studies had small homogenous samples containing largely stroke patients, affects the confidence in the validity of the findings and limits their generalizability. It was found to be difficult to obtain relevant literature with all participants over age 65. Therefore, studies were included that had participants under age 65, if the majority of subjects were over age 65. A strength of this review was that studies were included from three different continents and found generally similar results on the effects AFOs have on balance in the older adult population. This provides reasonable confidence in the external validity of the findings within a specific population of patients.

### Recommendations and implications for practice

The findings of this scoping review generally support that AFOs affect balance in older adults in a positive way. The observation that though restrictions to the natural movement of the ankle can occur through wearing AFOs, the AFOs effect on improving proprioception appeared to remedy any potential deficit. This is an important clinical consideration when prescribing AFOs to patients who may have sensory deficits, to ensure the AFO does not negatively impact the limitations their pathology imposes. This is pertinent information for healthcare providers who prescribe AFOs or work with AFO wearing individuals, and the evidence derived from this review indicates the relevance of a more in-depth examination of literature in this subject area.

This review did not display better balance outcomes for individuals wearing custom fabricated AFOs, suggesting that off-the-shelf, prefabricated AFOs may work sufficiently for certain patient populations. Custom AFOs are substantially more expensive than their prefabricated counterparts, so healthcare providers must give consideration to appropriate use of the healthcare resources available when prescribing AFOs.

The patient populations examined in this review were small samples of hemiplegic stroke and peripheral neuropathy patients. Investigation into the effects AFOs have on other older adult populations with more diverse pathologies is an important area for future study to help substantiate the evidence found to support the AFOs affect on balance in these two specific patient groups. Studies conducted with larger sample sizes in more diverse setting would aid in validating the findings of this review.

## CONCLUSION

The purpose of this review was to investigate the role AFOs play in affecting balance in community-dwelling older adults. Given the many dimensions that comprise the ability to balance, evidence was examined for consistencies amongst findings for which aspects of balance were most impacted by AFOs. The AFO was found to increase lateral stability, improve balance under static conditions, better control postural sway, and increase walking speed. The findings indicate that the AFO has a generally positive affect on balance in older adults with hemiplegia and peripheral neuropathy, and supports the use of AFOs in patient populations with sensory impairments to improve balance and walking speed. Investigation into the affects of AFOs on the balance of other pathologic patient populations and on balance during dynamic conditions warrants further research.

## DECLARATION OF CONFLICTING INTERESTS

The author has no conflicts of interest to declare.

## SOURCES OF SUPPORT

No funding was provided for this review.

## ETHICAL APPROVAL

Ethical approval was not needed for this study.

## APPENDIX (A)

Appendix (A): example database search strategy

**MEDLINE Search Strategy** (Literature search performed: February 2, 2020)

1. Orthotic devices/ or braces/

2. (AFO or ankle foot ortho*)

3. exp^#^ Postural balance/

4. Balance

5. exp Aged

6. older adult*

7. (Orthotic devices/ or braces/) or (AFO or ankle foot ortho*)

8. (exp Postural balance/) or (balance)

9. (exp Aged) or (older adult*)

10. ((Orthotic devices/ or braces/) or (AFO or ankle foot ortho*)) and ((exp Postural balance/) or (balance)) and ((exp Aged) or (older adult*))

**CINHAL Search Strategy** (Literature search performed: February 2, 2020)

1. (MH “Orthoses+”)

2. “ankle foot orthosis or afo or orthotic”

3. (MH “Balance, Postural”)

4. “balance”

5. (MH “Aged+)

6. “older adults”

7. ((MH “Orthoses+”)) OR (“ankle foot orthosis or afo or orthotic”)

8. ((MH “Balance, Postural”)) OR (“balance”)

9. ((MH “Aged+)) OR (“older adults”)

10. [((MH “Orthoses+”)) OR (“ankle foot orthosis or afo or orthotic”)] AND [((MH “Balance, Postural”)) OR (“balance”)] AND [((MH “Aged+)) OR (“older adults”)]

**EMBASE Search Strategy** (Literature search performed: February 2, 2020)

1. exp ankle foot orthosis/

2. (AFO or ankle foot ortho*)

3. exp balance impairment/

4. balance

5. exp aged/

6. older adults

7. (exp ankle foot orthosis/) OR ((AFO or ankle foot ortho*))

8. (exp balance impairment/) OR (balance)

9. (exp aged/) OR (older adults)

10. [(exp ankle foot orthosis/) OR ((AFO or ankle foot ortho*))] AND [(exp balance impairment/) OR (balance)] AND [(exp aged/) OR (older adults)]

**REHABDATA Search Strategy** (Literature search performed: February 2, 2020)

View Articles, including International Research, containing the exact phrase: ‘“ankle foot ortho*”‘, containing at least one of the word(s): ‘“balance”‘, where Abstract contains: [(older AND adult) OR (ankle AND foot) AND (ortho* AND balance)

# exp= explode - In database searches, the subject heading can be ‘exploded’ to include other more specific terms that are related to the initial subject heading in the search results.

## APPENDIX (B)

Appendix (B): Quality appraisal and rating of studies reviewed

**Table:**

**Author, Year**	**Neutrality Objectivity**	**Consistency Reliability**	**Applicability External Validity**	**Truth Value Internal Validity**	**Quality Rating ^a^**
Wang et al., 2019^27^	High	High	Moderate	Moderate	3
Chen et al., 2008^20^	High	High	Moderate	Moderate	3
Bigelow & Jackson, 2014^26^	Moderate	High	Low	High	3
Wang et al., 2005^22^	Moderate	High	Moderate	Moderate	2
Wang, et al., 2007^23^	Moderate	Moderate	Low	Moderate	2
Simons et al., 2009^19^	Moderate	Low	Low	Moderate	2
Yalla et al., 2014^28^	Low	Moderate	Low	Moderate	2
Nikamp et al., 2017^18^	Moderate	High	Moderate	Low	2
Dogan et al., 2011^25^	Low	Moderate	Low	Moderate	1
Cakar et al., 2010^24^	Low	Moderate	Low	Moderate	1
Chen et al., 1999^21^	Moderate	Low	Low	Low	1

a - Studies arranged in order of quality rating: 1 = weak; 2 = moderate; 3 = strong

## APPENDIX (C)

Appendix (C): data extraction table – characteristics of the included studies

**Table:**

**Author, Year**	**Aim of study/paper**	**Sample size/ characteristics**	**Study Design**	**AFO type & features**	**Balance measures**	**Main findings/ conclusions**	**Strengths and limitations**	**Article quality rating**
Bigelow & Jackson, 2014^26^	To observe the immediate effects of AFOs on balance and gait in individuals with peripheral neuropathy.	n=12 patients with peripheral neuropathy; convenience sample	Pre-/ post-test Quantitative Within-subjects comparison	Anterior shell carbon composite AFOs Prefabricated Bilateral	Force plate posturography^a^ • LOS • AP/ML sway + velocity Clinical tests • MiniBESTest • TUG • Gait speed	AFO has immediate improvements in static postural control but more variable responses during dynamic balance and gait. AFOs restrict AP LOS. No sig. difference in AFO conditions for clinical balance and gait assessments.	First time AFO wearers, given same amount of practice time Small sample Reduced ordering effect Did not use standard measures in dynamic balance tests	3
Cakar et al., 2010^24^	To investigate the relative effect of AFOs on balance and fall risk.	n=25 hemiplegic long duration stroke patients with spasticity	Pre-/ post-test Quantitative Within-subjects comparison	Thermoplastic posterior leaf spring AFO Prefabricated Unilateral	BBS Postural Stability test ^b^ Fall Risk test ^b^	AFO improved balance and provided fall risk reduction in hemiparetic patients with mild/moderate spasticity; no difference in AP/ML stability scores.	Used verified & valid test measures Excluded 2 outliner scores Distance from researcher Homogenous group of participants	1
Chen et al., 1999^21^	To evaluate the effects of an AFO on static and dynamic postural stability in hemiplegic patients.	n=24 hemiplegic patients; convenience sample	Pre-/ post-test Quantitative Within-subjects comparison	Thermoplastic anterior leaf-spring AFO Prefabricated Unilateral	Stabiloboard^d^ weight-shift AP/ML max. balance Postural sway & symmetry	Significant improvement in lateral weight shift and weight bearing through affected leg with AFO. No difference in postural sway, postural symmetry or AP max. balance range with AFO.	Poorly described study design; hard to replicate condition Small homogeneous convenience sample Dynamic postural stability was assumed Random assignment to test condition	1
Chen et al., 2008^20^	To evaluate the effects of an AFO on postural stability in stroke patients with hemiplegia.	n=21 patients with new onset stroke (<3mo.) hemiplegia + 10 healthy subjects	Pre-/ post-test Quantitative Cross sectional + control group comparison	Thermoplastic anterior leaf-spring AFO Prefabricated Unilateral	Postural stability ^c^ - Ankle strategy - Max stability - COG velocity	Anterior AFO may be used to assist early stage, hemiparetic stroke patients improve postural stability during stance by reducing COG velocity, lessening likelihood of falls.	Standardized test Conditions random Comparison group Single, 1-hour session of testing Only tested in static conditions	3
Dogan et al., 2011^25^	To investigate whether AFOs have an effect on stair climbing, balance and mobility while improving walking parameters.	n=51 hemiplegic stroke patients	Pre-/ post-test Quantitative Within-subjects comparison	Hinged AFO with 90° PF stop Custom made Unilateral	Ashburn Walking and Stair test TUG test BBS STREAM	AFOs improved balance and ambulation activities in hemiparetic subjects. All subject showed improvement in gait speed, balance and mobility with AFO use. No affect on stair climbing.	Large number of exclusion criteria Larger sample then most other studies Ceiling effects for some tests used Instrumentation effect Attrition effect	1
Nikamp et al., 2017^18^	To study 6-month effects of early/late provision of AFOs in (sub)acute stroke patients To look at group differences and affects on functional improvement overtime.	n=33 unilateral hemiplegic stroke patients max. 6 weeks post stroke Early (wk 1) n=16; Late (wk 9) n=17	RCT 6 mo. f/u Quantitative Parallel group comparison	3 types of non-articulated posterior leaf spring thermoplastic AFOs -rigid -semi-rigid -flexible Prefabricated Unilateral	10m walk test BBS FAC 6 min walk test TUG test Stairs test Barthel index Rivermead Mobility index	No 6-month differences in functional outcomes of providing AFOs at different times in early rehab after stroke. In general, both groups of AFO wearers improved over time	Registered RCT Underpowered Maturation effect - natural recovery post-stroke No control group Randomly assigned groups by 3rd party Learning occurred out of clinical setting Not possible to blind	2
Simons et al., 2009^19^	To examine effects of AFOs on functional balance, static and dynamic weight bearing asymmetry, and balance control in stroke patients.	n=20 hemiparetic stroke patients (at least 3 mo. post-stroke)	Pre-/ post-test Quantitative Within-subjects comparison with washout period	Various flexible and rigid AFO designs Patient had own AFO at study outset Custom made+ Prefabricated Unilateral	Functional tests - BBS - TUG - 10m walk - FAC - TBT Posturo-graphic tests -CAREN	AFO improved performance of functional tests, but had no effect on weight bearing asymmetry or dynamic balance. No effect on postural sway. Increased walking speed	Highly sophisticated CAREN system Washout period between trials Attrition effect Only measured AP not ML balance during perturbations	2
Wang et al., 2019^27^	To investigate effectiveness of AFOs on balance, fear of falling, and physical activity in older adults.	n=44 non-pathologic patients with fear of falling, or previous fall	RCT; Quantitative Longitudinal (6mo f/u)	Flexible anterior gauntlet AFO Custom made Bilateral	TUG AST Postural sway (COM, ankle, hip)	AFO + walking shoes improve balance compared to walking shoes alone and significantly reduces fear of falling. Increased lateral stability	Registered RCT Many exclusions Non-pathologic participants Underpowered Attrition effect- 25% dropout rate History effect	3
Wang et al., 2005^22^	To examine the effects of AFO on balance in patients with short and long duration hemiplegia.	n=103 subjects with long (>12mo)/ short(<6mo) duration unilateral hemiparesis SD n=42 LD n=61	Pre-/ post-test Quantitative between group comparison	Thermoplastic AFO Prefabricated Unilateral	Standing balance ^c^ - COG velocity, excursion, accuracy - LOS Sit-to-stand BBS 10m walk test	AFO improves symmetry in static + dynamic balance, and increases gait speed and cadence in subjects with short duration hemiparesis. Effects not observed for long duration subjects.	Largest sample size in data set No control group Cane used in walking trials Test sequence random Gait assessed by independent physiotherapist	2
Wang et al., 2007^23^	To assess changes in balance, and improvement in gait of hemiplegic subjects as a result of wearing an AFO	n=58 subjects with hemiparesis (within 6 mo. post-stroke)	Pre-/ post-test Quantitative Within-subjects comparison	Thermoplastic AFO Prefabricated Unilateral	Standing balance ^c^ - COG velocity and excursion - LOS Gait parameters” (time/distance)	AFO improved dynamic balance and increased walking speed in hemiparetic subjects. Increased lateral weight bearing on AFO side	Random test sequence Researcher performed tests Walking speed much faster than previous studies’ findings	2
Yalla et al., 2014^28^	To determine the immediate effect of AFOs on balance and functional reach distance in older adults	n=30 diabetic/ peripheral neuropathy-excluded hemiplegics	Pre-/ post-test Quantitative Within-subjects comparison	Flexible anterior gauntlet AFO Bilateral Custom made	Postural Sway^f^ - COM, ankle, hip Forward Reach test TUG	Wearing AFO reduced postural sway and improved lower extremity coordination in subjects without interfering with their ability to perform ADLs	Less restricted patient population than other studies Clear/consistent protocol Instrumentation effects - sensors vs. force plate used	2

LOS=Limit of stability; TUG=Timed Up and Go; AP= Anterior-posterior; ML=Medial-lateral; BBS=Berg Balance Scale; COG=Centre of gravity; PF=Plantar flexion; STREAM=Stroke rehabilitation assessment of movement; FAC=Functional Ambulation Categories; TBT=Timed Balance trial; AST=Alternate-Step Test; ADLs=Activities of daily living

a from BP5050 (Bertec Corporation, Columbus, OH, USA)

b from Biodex Balance System (Biodex Medical Systems, Shirley, NY, USA)

c from SMART Balance Master System (NeuroCom International, Inc., Clackamas, OR, USA)

d from Computer Dyno Graphy system (Market-USA Inc., Severna Park, MD, USA)

e from GAITRite system (CIR System Inc., Franklin, NJ, USA)

f from BalanSens™ Sensors (BioSensics LLC, Boston, USA)
